# Public awareness and knowledge of factors associated with dementia in China

**DOI:** 10.1186/s12889-020-09665-7

**Published:** 2020-10-17

**Authors:** Yong-Bo Zheng, Le Shi, Yi-Miao Gong, Xiao-Xiao Wang, Qing-Dong Lu, Jian-Yu Que, Muhammad Zahid Khan, Yan-Ping Bao, Lin Lu

**Affiliations:** 1 Peking University Sixth Hospital, Peking University Institute of Mental Health, NHC Key Laboratory of Mental Health (Peking University), National Clinical Research Center for Mental Disorders (Peking University Sixth Hospital), Beijing, China; 2 Peking-Tsinghua Center for Life Sciences and PKU-IDG/McGovern Institute for Brain Research, Peking University, Beijing, China; 3 Research Center of Clinical Epidemiology, Peking University Third Hospital, Beijing, China; 4 National Institute on Drug Dependence and Beijing Key Laboratory of Drug Dependence, Peking University, Beijing, China

**Keywords:** Public awareness, Understanding, Dementia, Factors, China

## Abstract

**Background:**

Dementia is a global public health priority. Many modifiable factors have been shown to influence the development of dementia, but these factors are not adequately known by the general public. This study aimed to assess public awareness of the factors that are associated with dementia in China.

**Methods:**

A cross-sectional study was conducted from May to October 2019 using an Internet-based questionnaire. Data on basic sociodemographic characteristics were collected, and the knowledge of risk and protective factors for dementia was investigated. Logistic regression analysis was performed to compare levels of the knowledge of factors associated with dementia across populations with different demographic characteristics.

**Results:**

Data from 3338 respondents were analyzed. The percentages of participants who accurately identified the risk factors of dementia were follows: 84.24% for negative affect, 65.07% for alcohol use, 56.68% for smoking, 48.74% for hypertension, and 42.66% for diabetes. The percentages of participants who accurately identified the protective factors for dementia were follows: 90.00% for exercise, 84.69% for social activity, 80.92% for intelligence games, 74.45% for reading, and 6.14% for antihypertensive or hypolipidemic drugs. The majority of Chinese people correctly recognized the role of lifestyle factors in the development of dementia but not medical factors. The levels of knowledge of the factors associated with dementia were significantly distinct across populations with different characteristics. The following sociodemographic characteristics were associated with more comprehensive knowledge of dementia risk and protective factors: women, young age, high education levels, nonmanual jobs, and contact with patients with dementia.

**Conclusions:**

Public awareness and knowledge of risk and protective factors for dementia in China are still insufficient. More efforts are needed to publicize information to reduce the risk of dementia.

## Background

Dementia is a global health priority worldwide. According to the 2016 Global Burden of Disease Study, approximately 43.8 million people lived with dementia worldwide in 2016 [1]. Moreover, this number is estimated to increase to 75 million by 2030, and the majority of these individuals will likely to be living in low- and middle-income countries [2]. Hence, preventing dementia has become crucial. Unfortunately, no treatments at present are available to cure dementia or alter its progressive course. Evidence shows that immutable factors (e.g., genetics and aging), lifestyle, and environmental variables are crucial in the development of dementia [3]. Notably, around one third of all dementia cases are likely to be caused by modifiable risk factors [4]**.** Thus, identifying and avoiding exposure to these modifiable risk factors may reduce the risk of having dementia [5, 6] and help reduce disease burden that is associated with dementia [7, 8]. Numerous studies have explored the factors influencing the development of dementia. Accumulating evidence suggests that people with unhealthy habits, such as smoking [9] and excessive alcohol intake [10], or having depression [11, 12] and chronic diseases (e.g., diabetes mellitus [13] and hypertension [14]), may have a higher risk of developing dementia. Leisure activities, including physical exercise [15], and social activities [16, 17], may reduce the risk of developing dementia. Although various risk and protective factors for dementia have been identified, they have not been adequately disseminated and recognized among the general public. For example, a systematic review assessed the knowledge and attitudes about dementia prevention and treatment and found that public knowledge about the critical role of modifying risk factors in reducing dementia risk remained inadequate, although this situation might improve over time [18]. A recent survey from the Netherlands showed that the majority of community-dwelling people were unaware of the relationship between lifestyle and brain health [19]. Most investigations of the knowledge and attitudes about dementia prevention were conducted in high-income countries. The levels of knowledge of the potential for dementia prevention among individuals who live in other countries, such as China, are largely unknown [18].

Life expectancies have increased sharply over recent decades, and dementia is a serious health problem in China. The age-standardized prevalence of dementia in China increased by 5.6% in China from 1990 to 2016, while the worldwide prevalence increased by only 1.7% [1]. The estimated number of cases of dementia and total annual costs that are associated with dementia in China were predicted to reach 24.25 million people and USD$114.2 billion in 2030 [20, 21]. The worldwide costs associated with Alzheimer’s dementia accounted for 1.09% of the global gross domestic product, whereas such costs accounted for 1.47% in China, indicating that the burden of dementia was even higher in China compared with the world average [22]. Thus, effective measures are needed to facilitate dementia prevention and reduce disease burden that is caused by dementia in China [23]. However, insufficient awareness and knowledge of the potential of dementia prevention among the Chinese public has been a substantial obstacle for dementia control and management [23]. Some studies evaluated the Chinese population’s overall understanding of dementia, suggesting that the recognition of dementia needed to be improved [24, 25]. The awareness of specific protective and risk factors associated with dementia among the Chinese population is inadequate. To identify specific target populations and develop strategies for dementia prevention, a better understanding of these factors is needed among populations with different sociodemographic backgrounds.

## Methods

### Study design and participants

An online questionnaire that evaluated general knowledge of dementia was disseminated via WeChat (i.e., a social media outlet widely used in China) from May 27, 2019, to October 6, 2019. Chinese individuals who lived in mainland China were invited to complete the questionnaire. The recruitment location of this online survey was not limited, and individuals across the nation had access to the survey. Participation in the study was voluntary, and the information collected was anonymous. The participants included in this survey met the following criteria: (1) Wechat users; (2) voluntary to participate in the survey; and (3) provided informed consent. The study was approved by the ethics committee of Peking University Sixth Hospital (Institute of Mental Health), and the approval number was 2020-4-9-1. Informed consent was obtained online before the respondents began the questionnaire. Only participants who provided informed consent were able to participate in the study. Teenagers aged between 8 and 18 were approved by the ethics committee to voluntarily participate in the study without parental consent. A total of 3436 participants submitted the questionnaire. Incomplete questionnaires were excluded. Finally, 3338 questionnaires from 32 of 34 province-level regions in China were analyzed.

### Questionnaire content

The questionnaire was written in Chinese and included two parts: (1) basic sociodemographic information, including sex, age, education level, type of job [nonmanual work (managers, etc.), manual work (farmers, etc.) and retired] [26], income, type of residence (city, town, and rural areas) [27], and whether the respondent had contact with anyone who lived with dementia, and (2) the following multiple-choice questions: “Which factors do you think can increase the risk of dementia?” and “Which factors do you do think can reduce the risk of dementia?” The questions had the following response options: exercise, smoking, alcohol use, reading, intelligence games, social activity, hypertension, diabetes, negative affect (e.g., anxiety and depression), antihypertensive or hypolipidemic drugs, and “none of the factors mentioned above.” The aforementioned factors were divided into two categories: lifestyle factors (smoking, alcohol use, and leisure activities) and medical factors (hypertension, diabetes, negative affect, and antihypertensive or hypolipidemic drugs) [28]. Leisure activities included exercise, reading, intelligence games, and social activity. Among the options, hypertension, diabetes, negative affect, smoking, and alcohol use were regarded as risk factors based on previous findings, and the other options were protective factors. The questionnaire took approximately 3 min to complete, and detailed information of the questionnaire was listed in Supplementary Table 1.

### Statistical analysis

Frequency distributions of all sociodemographic characteristics and the proportion of participants who identified each item as a risk or protective factor were calculated. Descriptive statistics were used to present sex-specific demographic characteristics, and χ2 tests were used to compare the demographic data among male and female participants. Multiple logistic regression analysis with all factors entered was used to compare the knowledge of factors for each item, stratified by demographic variables. In addition, sex-specific differences in the association between demographic characteristics and knowledge of factors for dementia were further compared by performing multiple logistic regression analysis. The adjusted odds ratios (AORs) and 95% confidence intervals (CIs) were presented. SPSS 22 software (SPSS, Chicago, IL, USA) was used to analyze the data. Values of *p* < 0.05 were considered statistically significant.

## Results

### Characteristics of the participants

Data from 3338 eligible samples were analyzed in the present study. Table 1 shows the demographic characteristics of the respondents (1154 males and 2184 females). The average age was 39.23 ± 12.50 years, ranging from 15 to 97 years. The proportions of different education levels, including primary school or illiteracy, middle school, college or university, and postgraduate education, were 1.71%, 18.51%, 50.00%, and 29.78%, respectively. A majority of the participants were nonmanual workers (77.29%) with an income of 2000–10,000 yuan per month (66.12%) and lived in cities (86.70%). Nearly one third of the participants reported that they previously had contact with someone with dementia. The demographic characteristics of sex-specific participants are presented in Supplementary Table 2. Age, type of job, and income were significantly different between male and female participants.

**Table 1:** Characteristics of the participants

	Number	Weighted proportion
**Sex**		
Men	1154	34.57%
Women	2184	65.43%
**Age (years)**		
< 40	1845	55.27%
40–65	1387	41.55%
≥ 65	106	3.18%
**Education level (years)**		
Primary school or illiteracy (≤ 6)	57	1.71%
Middle or high school (6–12)	618	18.51%
College or university (12–16)	1669	50.00%
Postgraduate (≥ 16)	994	29.78%
**Type of job**		
Nonmanual	2580	77.29%
Manual	405	12.13%
Retired	353	10.58%
**Income group (yuan/month)**^**1**^		
0–2000	366	10.9 6%
2000–5000	978	29.30%
5000–10,000	1229	36.82%
> 10,000	765	22.92%
**Type of residence**^**2**^		
City	2894	86.70%
Town	290	8.69%
Rural area	154	4.61%
**Dementia contact**		
Yes	1101	32.98%
No	1676	50.21%
Unclear	561	16.81%

^1^ 1 yuan ≈ 0.14 dollar. ^2^ “City” refers to the centre area of the big city. “Town” is defined as all the surrounding districts and county cities. “Rural area” mainly involves remote residential villages

### Awareness and understanding of risk factors for dementia

**Fig. 1:**
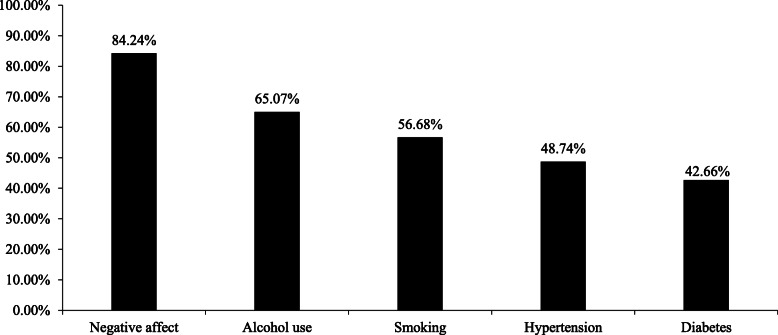
Proportion of participants who identified each risk factor associated with dementia

The proportions of each item identified by the respondents as a risk factor for dementia are presented in Fig. 1. Most of the participants could correctly recognize at least one risk factor, but 5.51% of them were unable to correctly recognize any single risk factor. The percentage of the participants who accurately identified the following risk factors for dementia was 84.24% for negative affect, 65.07% for alcohol use, 56.68% for smoking, 48.74% for hypertension, and 42.66% for diabetes.

Next the relationship between sociodemographic characteristics and knowledge of risk factors for dementia were assessed using multiple logistic regression analysis (Table 2). Compared with men, more women believed that negative affect (AOR = 1.68, 95% CI = 1.37–2.05) was a risk factor for dementia. The sex-specific analysis showed that female participants with different education levels and types of job as well as whether they had contact with dementia exhibited a distinct knowledge of risk factors for dementia; however, no demographic factors were associated with all five risk factors among male participants (Supplementary Table 3). Middle-aged individuals (aged 40–65 years) were significantly less likely to identify the contribution of negative affect (AOR = 0.69, 95% CI = 0.56–0.86), alcohol use (AOR = 0.83, 95% CI = 0.71–0.99), and smoking (AOR = 0.67, 95% CI = 0.57–0.79) to the progression of dementia compared with younger individuals (aged < 40 years). Additionally, the group with a higher level of education (college, university, and postgraduate) had a better understanding of all five dementia risk factors for dementia compared with the group with a low education level (primary school or illiteracy) group.

**Table 2:** Demographic and social factors associated with the knowledge of risk factors for dementia

	Negative affect			Alcohol use			Smoking			Hypertension			Diabetes		
	%	AOR	95% CI	%	AOR	95% CI	%	AOR	95% CI	%	AOR	95% CI	%	AOR	95% CI
**Sex**															
Men	80.80	1		64.60	1		55.10	1		48.70	1		41.80	1	
Women	86.10	1.68*	1.37–2.05	65.30	1.10	0.94–1.29	57.50	1.14	0.98–1.33	48.80	1.06	0.91–1.23	43.10	1.10	0.95–1.28
**Age (years)**															
< 40	87.70	1		69.40	1		63.30	1		52.00	1		45.70	1	
40–65	80.00	0.69*	0.56–0.86	60.60	0.83*	0.71–0.99	49.20	0.67*	0.57–0.79	45.60	0.89	0.76–1.05	39.70	0.92	0.78–1.08
≥ 65	79.20	1.19	0.67–2.11	48.10	0.79	0.50–1.25	38.70	0.64	0.40–1.02	34.00	0.80	0.50–1.29	29.20	0.90	0.55–1.47
**Education level (years)**															
Primary school or illiteracy (≤ 6)	59.60	1		38.60	1		31.60	1		29.80	1		17.50	1	
Middle or high school (6–12)	73.50	2.02*	1.13–3.60	49.70	1.58	0.89–2.78	40.30	1.41	0.78–2.54	32.70	1.10	0.60–2.01	26.50	1.57	0.77–3.21
College or university (12–16)	86.80	3.78*	2.07–6.89	67.20	2.61*	1.47–4.66	59.30	2.25*	1.24–4.10	50.90	1.99*	1.08–3.64	44.20	2.81*	1.37–5.75
Postgraduate (≥ 16)	88.10	3.69*	1.95–6.97	72.60	3.13*	1.72–5.70	63.90	2.47*	1.33–4.57	56.10	2.24*	1.20–4.17	51.60	3.51*	1.69–7.30
**Type of job**															
Nonmanual	86.70	1		69.20	1		61.00	1		52.40	1		46.60	1	
Manual	78.50	0.95	0.69–1.31	54.60	0.78	0.61–1.00	45.20	0.75*	0.59–0.96	38.30	0.84	0.66–1.08	30.10	0.78	0.60–1.01
Retired	72.80	0.55*	0.39–0.76	46.70	0.56*	0.43–0.74	38.20	0.60*	0.45–0.79	34.30	0.67*	0.51–0.90	28.60	0.62*	0.46–0.84
**Income groups (yuan/month)**															
0–2000	80.60	1		59.60	1		54.60	1		42.30	1		37.40	1	
2000–5000	82.40	1.09	0.79–1.51	60.80	1.08	0.83–1.40	53.00	0.95	0.73–1.23	45.20	1.14	0.88–1.47	38.80	1.06	0.82–1.38
5000–10,000	84.40	0.96	0.69–1.34	67.20	1.13	0.87–1.47	58.90	0.95	0.74–1.23	48.90	1.06	0.83–1.37	43.50	1.01	0.78–1.30
> 10,000	88.10	1.35	0.92–1.97	69.70	1.13	0.85–1.51	58.80	0.88	0.66–1.16	56.10	1.30	0.99–1.71	48.80	1.11	0.84–1.47
**Type of residence**															
City	87.70	1		65.80	1		58.10	1		49.90	1		44.20	1	
Town	79.70	0.90	0.65–1.25	62.10	1.13	0.86–1.47	48.30	0.84	0.65–1.09	42.10	0.93	0.72–1.20	34.80	0.88	0.67–1.15
Rural area	75.30	0.79	0.52–1.21	57.10	1.09	0.76–1.56	46.10	0.86	0.60–1.23	39.00	0.97	0.68–1.39	27.90	0.76	0.52–1.12
**Dementia contact**															
Yes	85.90	1		69.10	1		59.20	1		54.20	1		48.50	1	
No	82.80	0.73*	0.59–0.91	62.90	0.72*	0.61–0.86	55.00	0.77*	0.66–0.91	45.00	0.67*	0.57–0.78	38.10	0.63*	0.54–0.74
Unclear	85.20	1.00	0.74–1.35	63.60	0.82	0.65–1.02	56.70	0.91	0.73–1.12	49.20	0.86	0.70–1.06	44.70	0.92	0.74–1.13

*Abbreviation AOR* adjusted odds ratio. *p < 0.05

Besides sex, age, and education level, the type of job, and contact with people with dementia also influenced the awareness of risk factors for dementia. Compared with nonmanual workers, manual workers had a poorer understanding of the relationship between smoking and dementia (AOR = 0.75, 95% CI = 0.59–0.96), and retired people had a lower understanding of all dementia risk factors for dementia. Respondents who were never in contact with patients with dementia were less likely to realize the roles that negative affect (AOR = 0.73, 95% CI = 0.59–0.91), alcohol use (AOR = 0.72, 95% CI = 0.61–0.86), smoking (AOR = 0.77, 95% CI = 0.66–0.91), hypertension (AOR = 0.67, 95% CI = 0.57-0.78) and diabetes (AOR = 0.63, 95% CI = 0.54–0.74) played in the development and progression of dementia compared with those were in contact with patients with dementia. The awareness of risk factors for dementia was not influenced by income or type of residence.

### Awareness and understanding of protective factors for dementia

**Fig. 2:**
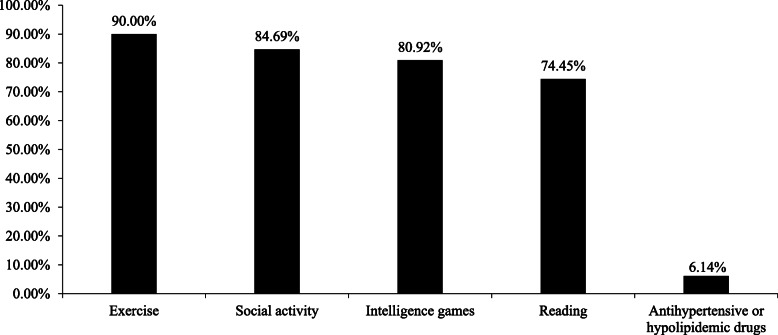
Proportion of participants who identified each protective factor associated with dementia

Figure 2 shows the proportions of participants who identified each item as a protective factor for dementia. The proportion of participants who chose none of the five items as protective factors was 4.79%. Most of the respondents correctly recognized exercise (90.00%), social activity (84.69%), intelligence games (80.92%), and reading (74.45%) as protective factors. However, only 6.14% of the individuals were aware that taking antihypertensive or hypolipidemic drugs was beneficial for delaying the onset of dementia.

Multiple logistic regression analysis was used to explore the effects of sociodemographic characteristics on the understanding of protective factors for dementia (Table 3). Sex, age, education level, type of job, and contact with patients with dementia had distinct influences on the awareness of protective factors for dementia. Compared with men, women knew more about the roles of exercise (AOR = 1.33, 95% CI = 1.04–1.71), social activity (AOR = 1.92, 95% CI = 1.57–2.35), intelligence games (AOR = 2.21, 95% CI = 1.82–2.69), and reading (AOR = 1.96, 95% CI = 1.65–2.33) in preventing dementia. Age, education level, type of job, and whether the individuals had contact with patients with dementia were associated with the knowledge of at least one protective factor for dementia in both male and female participants (Supplementary Table 4). Compared with younger individuals (aged < 40 years), middle-aged participants and elderly individuals were less aware that exercise (aged 40–65 years: AOR = 0.60, 95% CI = 0.45–0.79; aged ≥65 years: AOR = 0.41, 95% CI = 0.23–0.72), intelligence games (aged 40–65 years: AOR = 0.47, 95% CI = 0.38–0.58; aged ≥65 years: AOR = 0.43, 95% CI = 0.26–0.72), and reading (age 40–65 years: AOR = 0.79, 95% CI = 0.65–0.95; aged ≥65 years: AOR = 0.51, 95% CI = 0.32–0.83) were protective factors. People with a higher level of education had a better understanding of lifestyle variables that were protective factors for dementia, with the exception of antihypertensive and hypolipidemic drugs. Compared with nonmanual workers, manual workers had lower knowledge that exercise (AOR = 0.64, 95% CI = 0.45–0.92) and reading (AOR = 0.70, 95% CI = 0.53–0.91) were protective factors. Individuals who had contact with patients with dementia had a better understanding that social activity (AOR = 0.78, 95% CI = 0.62–0.98) and reading (AOR = 0.81, 95% CI = 0.67–0.98) were protective factors for dementia compared with individuals who had no contact with patients with dementia. Only people who had contact with patients who had dementia knew that antihypertensive and hypolipidemic drugs might help prevent dementia (AOR = 0.49, 95% CI = 0.35–0.67). The income and type of residence did not influence the awareness of protective factors for dementia as that of risk factors.

**Table 3:** Demographic and social factors associated with the knowledge of protective factors for dementia

	Exercise			Social activity			Intelligence games			Reading			Antihypertensive or hypolipidemic drugs		
	%	AOR	95% CI	%	AOR	95% CI	%	AOR	95% CI	%	AOR	95% CI	%	AOR	95% CI
**Sex**															
Men	89.17	1		79.55	1		74.61	1		66.72	1		6.41	1	
Women	90.43	1.33*	1.04–1.71	87.41	1.92*	1.57–2.35	84.25	2.21*	1.82–2.69	78.66	1.96*	1.65–2.33	6.00	0.91	0.68–1.24
**Age (years)**															
< 40	93.60	1		87.37	1		88.40	1		78.86	1		6.88	1	
40–65	86.52	0.60*	0.45–0.79	82.26	0.82	0.65–1.02	72.60	0.47*	0.38–0.58	70.37	0.79*	0.65–0.95	5.26	0.77	0.55–1.07
≥ 65	72.64	0.41*	0.23–0.72	69.81	0.54	0.32–0.92	59.43	0.43*	0.26–0.72	53.77	0.51*	0.32–0.83	4.72	0.30	0.30–2.41
**Education level (years)**															
Primary school or illiteracy (≤ 6)	64.91	1		56.14	1		35.09	1		40.35	1		3.51	1	
Middle or high school (6–12)	80.42	2.26*	1.23–4.13	73.30	2.12*	1.19–3.76	60.52	3.19*	1.76–5.80	55.50	1.82*	1.03–3.21	3.88	0.99	0.22–4.37
College or university (12–16)	91.19	3.62*	1.91–6.85	85.68	3.70*	2.04–6.72	84.66	7.75*	4.18–14.36	77.77	4.14*	2.30–7.43	6.47	1.40	0.32–6.20
Postgraduate (≥ 16)	95.37	5.47*	2.68–11.15	91.75	6.37*	3.35–12.12	89.94	11.21*	5.82–21.59	82.90	5.38*	2.92–9.93	7.14	1.49	0.33–6.77
**Type of job**															
Nonmanual	93.02	1		87.36	1		85.54	1		78.57	1		6.67	1	
Manual	82.47	0.64*	0.45–0.92	74.07	0.75	0.56–1.02	68.89	0.85	0.63–1.14	58.77	0.70*	0.53–0.91	4.20	0.76	0.43–1.35
Retired	76.49	0.53*	0.36–0.77	77.34	0.86	0.60–1.23	60.91	0.57*	0.42–0.79	63.17	0.80	0.59–1.08	4.53	0.82	0.43–1.54
**Income groups (yuan/month)**															
0–2000	86.61	1		80.33	1		75.68	1		70.49	1		6.01	1	
2000–5000	86.50	1.02	0.70–1.48	82.31	1.21	0.87–1.66	75.56	1.01	0.73–1.38	71.78	1.03	0.78–1.38	5.62	1.00	0.59–1.70
5000–10,000	90.97	1.15	0.77–1.72	85.68	1.18	0.85–1.65	84.95	1.29	0.93–1.80	76.00	0.95	0.71–1.27	6.67	0.99	0.60–1.64
> 10,000	94.51	1.61	0.99–2.63	88.24	1.31	0.90–1.92	83.79	1.04	0.72–1.51	77.65	0.98	0.71–1.36	6.01	0.82	0.47–1.43
**Type of residence**															
City	90.50	1		85.83	1		82.38	1		76.09	1		6.46	1	
Town	88.62	1.32	0.87–1.98	77.93	0.89	0.64–1.22	74.83	1.07	0.78–1.46	68.28	1.02	0.77–1.36	3.10	0.51	0.25–1.03
Rural area	83.12	1.01	0.61–1.66	75.97	1.04	0.68–1.61	64.94	0.78	0.52–1.18	57.14	0.81	0.56–1.18	5.84	1.11	0.53–2.33
**Dementia contact**															
Yes	91.19	1		87.19	1		82.02	1		77.20	1		8.27	1	
No	90.21	0.84	0.64–1.11	84.19	0.78*	0.62–0.98	81.68	0.89	0.72–1.10	73.75	0.81*	0.67–0.98	4.47	0.49*	0.35–0.67
Unclear	86.99	0.71*	0.50–0.99	81.28	0.70*	0.53–0.94	76.47	0.74*	0.56–0.97	71.66	0.81	0.64–1.04	6.95	0.82	0.55–1.21

*Abbreviation AOR* adjusted odds ratio. *p < 0.05

## Discussion

The present study investigated public knowledge and awareness of factors associated with dementia in China using a relatively large sample. Most people could correctly recognize evidence-based risk and protective factors. However, a majority of respondents were unaware of the role of medical factors in the development of dementia. Additionally, the understanding of factors related to dementia was significantly associated with sociodemographic variables, such as sex, age, education level, type of job, and contact with patients with dementia. These findings underscored the necessity to invest more time and resources to promote the public knowledge of dementia in China and develop different strategies for people with different backgrounds.

Previous studies suggested that the knowledge of the possibility that dementia could be prevented remained poor in general. A few studies conducted in China focused mainly on the recognition of prodromal symptoms of dementia [18, 24]. The present results suggested that the overall understanding of factors associated with dementia among the Chinese population was more comprehensive than we initially believed based on similar studies in other countries [19, 29, 30]. This finding might have two explanations. First, the respondents in the present study had a relatively high level of education and might better comprehend and implement public health messages about dementia risk reduction. Second, an online questionnaire was used and people were invited to participate through the Internet, suggesting that the respondents might have better access to information about brain health compared with the general public. Future studies should compare the public awareness of dementia between China and other countries and evaluate the role of the Internet in disseminating brain health information.

Two categories of factors (lifestyle factors and medical factors) associated with dementia were used as response options in the present study [28]. Most of the respondents correctly recognized the relationship between lifestyle factors (e.g., alcohol use, smoking, and leisure activities) and dementia, but they often underestimated the contribution of medical conditions and certain medications to the development of dementia. This might be explained by the fact that the publicity of disease prevention focused mainly on lifestyle elements [31, 32], thus neglecting the fact that chronic diseases, including hypertension and diabetes, were risk factors for dementia [33]. Moreover, the critical roles of medications, such as antihypertensive and hypolipidemic drugs, by adequately controlling chronic diseases in dementia risk reduction are often neglected. Previous studies indicated that several reasons, including concerns about the side effects, high financial burden and inadequate health literacy, might lead to poor medication adherence [34, 35]. Other studies also found that people had less knowledge about the role of cardiovascular disease management in the development of dementia [19, 36]. Thus, medical factors associated with the risk reduction of dementia need to be popularized in the general public.

Several sociodemographic characteristics, including female sex, age < 40 years, a high level of education, nonmanual job, and having contact with patients with dementia, were associated with an extensive understanding of the modifiable risk and protective factors for dementia, which is partially consistent with previous surveys conducted in China [25, 37]. Women often have a better understanding of dementia in both China and other countries [25, 36, 38], and are more likely to adhere to guidelines [39]. Moreover, the knowledge of influencing factors for dementia was significantly affected by education level, type of job, and whether contact with patients with dementia in female participants; while these sociodemographic characteristics had a mild effect on the knowledge of factors for dementia among male participants. Individuals with a high level of education or previous contact with patients with dementia might have a higher probability of accessing information about dementia. In the present study and another survey conducted in Asia [40], younger people had more knowledge about dementia compared with middle-aged and elderly individuals. These findings suggested the need to develop different approaches for different populations to disseminate knowledge about dementia. In the present study, no significant influence of income or type of residence was found, which was contrary to previous findings that people with low income or living in rural areas were less likely to understand that the risk of dementia could be reduced [24, 37]. Future studies should recruit more individuals to confirm the results.

The strengths of the present study included (1) investigating the knowledge of risk and protective factors for dementia in a relatively large sample of the Chinese population and (2) exploring demographic characteristics associated with the level of knowledge of dementia. Informing the public about the modifiable risk and protective factors may help reduce the incidence of dementia.

The study also had several limitations. First, selection bias might have been unavoidable because of the use of an Internet-based social media application (i.e., WeChat). In this study, the participants were generally young, highly educated, and with high income levels, thus limiting the representativeness of the sample. Second, simple idioms were used instead of professional terms so that the response options could be more easily understood by the general public, which might have caused some ambiguity. Moreover, standardized scales should be used to measure the willingness of modifying lifestyle risk factors for dementia [41]. Third, the risk and protective factors mentioned in the survey were not comprehensive, and other modifiable factors associated with dementia (e.g., less education, social isolation, and obesity) were not assessed [4].

## Conclusions

In summary, the present Internet-based survey demonstrated substantial deficits in the public knowledge of modifiable factors for dementia in China. People were well aware of the role of lifestyle factors in dementia risk reduction, but the knowledge of medical factors in developing dementia was less understood. In addition, people with different characteristics might have distinct awareness of dementia risk. These findings indicated the importance of disseminating information about dementia in China and educating the public about the role of modifiable risk and preventive factors. More information about dementia risk reduction should be delivered to the public, and different promotion strategies are needed to achieve prevention.

## Supplementary information

**Additional file 1: Supplementary Table S1.** Summary of the questionnaire used in this study. **Supplementary Table S2.** Demographic characteristics among male and female participants. **Supplementary Table S3.** Sex differences in the association between demographic characteristics and the knowledge of risk factors for dementia. **Supplementary Table S4.** Sex differences in the association between demographic characteristics and the knowledge of protective factors for dementia.

## Data Availability

The primary data set collected from households and analyzed during the current study is available from the corresponding author.

## Abbreviations

AOR: Adjusted odds ratio
CI: Confidence interval
