# Supplementary material for: Public awareness and knowledge of factors associated with dementia in China
Source: BMC Public Health. 2020 Oct 17;20:1567. doi: 10.1186/s12889-020-09665-7 (PMC7568826; doi:10.1186/s12889-020-09665-7)
Supplement: Supplementary file 1 — Additional file 1: Supplementary Table S1. Summary of the questionnaire used in this study. Supplementary Table S2. Demographic characteristics among male and female participants. Supplementary Table S3. Sex differences in the association between demographic characteristics and the knowledge of risk factors for dementia. Supplementary Table S4. Sex differences in the association between demographic characteristics and the knowledge of protective factors for dementia. [file 12889_2020_9665_MOESM1_ESM.docx]

**Supplementary Table 1. Summary of the questionnaire used in this study.**

| **Questions** | **Types** | **Choices** |
| --- | --- | --- |
| 1. Please select your gender: | Single choice | Male/Female |
| 2. Please fill in your age (years): | Blank | / |
| 3. Please select your highest level of education: | Single choice | Illiteracy/Primary school/Middle school/High school/ College or university/Postgraduate |
| 4. Please select your type of job: | Single choice | Nonmanual/Manual/Retired |
| 5. Do you perform shift work? | Single choice | Yes/No |
| 6. Please select your monthly income (yuan): | Single choice | 0-2000/2000-5000/5000-10000/> 10000 |
| 7. Please select your type of residence: | Single choice | City/Town/Rural area |
| 8. Please select your typical sleeping time every day in the past one month (hours): | Single choice | < 5/5-6/6-7/7-8/8-9/> 9 |
| 9. Please select your self-reported sleep quality in the past one month (hours): | Single choice | Very good/Pretty good/Pretty bad/ Very bad |
| 10.Please select the type of neurological or psychiatric disorder you have been diagnosed: | Multiple choice | Insomnia/Sleep apnea hypopnea syndrome/Hypersomnolence/Restless leg syndrome/Major depressive disorder/Anxiety disorder/Other neurological or psychiatric disorders (Please fill in)/No |
| 11. Do you think poor sleep will increase the risk of dementia? | Single choice | Yes/No/Unclear |
| 12. Do you think taking sleeping pills will increase the risk of dementia? | Single choice | Yes/No/Unclear |
| 13. If poor sleep increases the risk of dementia, would you like to find a way to improve sleep quality? | Single choice | Yes/No/Unclear |
| 14. If you have sleep disturbances, what kind of methods would you like to take to improve your sleep quality? (this depends on "Yes" or "Unclear" being checked in the last question) | Multiple choice | Regular life/Strengthened exercise/ Psychotherapy/ Medication/Others (Please fill in) |
| 15. If there was a drug that could improve sleep quality and reduce the risk of/or prevent dementia, would you like to take it? | Single choice | Yes/No/Unclear |
| 16. Which factors do you think can increase the risk of dementia? | Multiple choice | Exercise/Smoking/Alcohol use/Reading/ Intelligence games/Social activity/Hypertension/ Diabetes/ Negative affect (e.g., anxiety and depression)/ Antihypertensive or hypolipidemic drugs/ None of the factors mentioned above |
| 17. Which factors do you think can reduce the risk of dementia? | Multiple choice | Exercise/Smoking/Alcohol use/Reading/ Intelligence games/Social activity/Hypertension/ Diabetes/ Negative affect (e.g., anxiety and depression)/ Antihypertensive or hypolipidemic drugs/ None of the factors mentioned above |
| 18. What kind of ways do you like to obtain knowledge about dementia? | Multiple choice | Not willing/Television and Internet (e.g., WeChat, Weibo, online video, etc.)/Books, magazines or newspaper/ Lecture on medication, or doctor consultation service/Others (Please fill in) |
| 19. Have you ever had contact with anyone who has lived with dementia? | Single choice | Yes/No/Unclear |

**Supplementary Table 2. Demographic characteristics among male and female participants.**

|  | **Male (%)** | **Female (%)** | ***p* Value** |
| --- | --- | --- | --- |
| **Overall** | 1154 | 2184 |  |
| **Age (years)** |  |  | 0.03* |
| < 40 | 661 (57.3) | 1184 (54.2) |  |
| 40-65 | 448 (38.8) | 939 (43.0) |  |
| ≥ 65 | 45 (3.9) | 61 (2.8) |  |
| **Education level (years)** |  |  | 0.35 |
| Primary school or illiteracy (≤ 6) | 15 (1.3) | 42 (1.9) |  |
| Middle or high school (6-12) | 216 (18.7) | 402 (18,4) |  |
| College or university (12-16) | 564 (48.9) | 1105 (50.6) |  |
| Postgraduate (≥ 16) | 359 (31.1) | 635 (29.1) |  |
| **Type of job** |  |  | ＜0.001* |
| Nonmanual | 935 (81.0) | 1645 (63.8) |  |
| Manual | 143 (12.4) | 262 (12.0) |  |
| Retired | 76 (6.6) | 277 (12.7) |  |
| **Income group (yuan/month) ^1^** |  |  | ＜0.001* |
| 0-2000 | 108 (9.4) | 258 (11.8) |  |
| 2000-5000 | 260 (22.5) | 718 (32.9) |  |
| 5000-10000 | 496 (35.2) | 823 (37.7) |  |
| > 10000 | 380 (32.9) | 385 (17.6) |  |
| **Type of residence ^2^** |  |  | 0.15 |
| City | 984 (85.3) | 1910 (87.5) |  |
| Town | 107 (9.3) | 183 (8.4) |  |
| Rural area | 63 (5.5) | 91 (4.2) |  |
| **Dementia contact** |  |  | 0.71 |
| Yes | 371 (32.1) | 730 (33.4) |  |
| No | 583 (50.5) | 1093 (50.0) |  |
| Unclear | 200 (17.3) | 361 (16.5) |  |

**Supplementary Table 3. Sex differences in the association between demographic characteristics and the knowledge of** **risk factors for dementia.**

|  | **Negative affect** | | | | **Alcohol use** | | | | | **Smoking** | | | | | **Hypertension** | | | | | **Diabetes** | | | | |
| --- | --- | --- | --- | --- | --- | --- | --- | --- | --- | --- | --- | --- | --- | --- | --- | --- | --- | --- | --- | --- | --- | --- | --- | --- |
| **Gender** | **Male** | | **Female** | | **Male** | | **Female** | | | **Male** | | **Female** | | | **Male** | | **Female** | | | **Male** | | **Female** | | |
|  | AOR | 95% CI | AOR | 95% CI | AOR | 95% CI | | AOR | 95% CI | AOR | 95% CI | | AOR | 95% CI | AOR | 95% CI | | AOR | 95% CI | AOR | 95% CI | | AOR | 95% CI |
| **Age (years)** |  |  |  |  |  |  | |  |  |  |  | |  |  |  |  | |  |  |  |  | |  |  |
| < 40 | 1.00 |  | 1.00 |  | 1.00 |  | | 1.00 |  | 1.00 |  | | 1.00 |  | 1.00 |  | | 1.00 |  | 1.00 |  | | 1.00 |  |
| 40-65 | 0.60* | 0.43-0.84 | 0.79 | 0.58-1.07 | 0.83 | 0.63-1.10 | | 0.82 | 0.67-1.02 | 0.64* | 0.49-0.83 | | 0.68* | 0.56-0.83 | 0.95 | 0.73-1.24 | | 0.87 | 0.71-1.06 | 0.92 | 0.70-1.21 | | 0.91 | 0.75-1.12 |
| ≥ 65 | 0.85 | 0.33-2.20 | 1.20 | 0.56-2.59 | 0.61 | 0.26-1.39 | | 0.94 | 0.53-1.70 | 0.47 | 0.20-1.10 | | 0.71 | 0.40-1.26 | 0.45 | 0.18-1.09 | | 1.02 | 0.57-1.84 | 0.53 | 0.21-1.32 | | 1.05 | 0.57-1.92 |
| **Education level (years)** |  |  |  |  |  |  | |  |  |  |  | |  |  |  |  | |  |  |  |  | |  |  |
| Primary school or illiteracy (≤ 6) | 1.00 |  | 1.00 |  | 1.00 |  | | 1.00 |  | 1.00 |  | | 1.00 |  | 1.00 |  | | 1.00 |  | 1.00 |  | | 1.00 |  |
| Middle or high school (6-12) | 2.36 | 0.79-7.09 | 2.03* | 1.01-4.07 | 1.02 | 0.35-3.00 | | 1.86 | 0.95-3.67 | 0.77 | 0.26-2.29 | | 1.80 | 0.87-3.75 | 0.93 | 0.30-2.92 | | 1.14 | 0.55-2.33 | 0.92 | 0.28-3.09 | | 1.94 | 0.79-4.81 |
| College or university (12-16) | 3.22* | 1.04-9.95 | 4.55* | 2.19-9.48 | 1.59 | 0.53-4.77 | | 3.13* | 1.57-6.25 | 1.16 | 0.39-3.52 | | 2.87* | 1.37-6.02 | 1.50 | 0.48-4.76 | | 2.15* | 1.04-4.44 | 1.39 | 0.41-4.71 | | 3.78* | 1.52-9.39 |
| Postgraduate (≥ 16) | 3.45* | 1.07-11.16 | 4.22* | 1.93-9.24 | 1.73 | 0.56-5.32 | | 4.03* | 1.96-8.27 | 1.33 | 0.43-4.24 | | 3.09* | 1.44-6.64 | 1.69 | 0.52-5.48 | | 2.43* | 1.15-5.13 | 1.94 | 0.56-6.71 | | 4.45* | 1.76-11.25 |
| **Type of job** |  |  |  |  |  |  | |  |  |  |  | |  |  |  |  | |  |  |  |  | |  |  |
| Nonmanual | 1.00 |  | 1.00 |  | 1.00 |  | | 1.00 |  | 1.00 |  | | 1.00 |  | 1.00 |  | | 1.00 |  | 1.00 |  | | 1.00 |  |
| Manual | 0.78 | 0.48-1.28 | 1.14 | 0.74-1.76 | 0.54* | 0.35-0.82 | | 0.92 | 0.68-1.26 | 0.71 | 0.46-1.08 | | 0.76 | 0.56-1.03 | 0.74 | 0.48-1.14 | | 0.90 | 0.67-1.23 | 0.86 | 0.55-1.35 | | 0.74 | 0.54-1.02 |
| Retired | 0.84 | 0.41-1.73 | 0.50* | 0.34-0.74 | 0.44* | 0.23-0.84 | | 0.63* | 0.46-0.87 | 0.61 | 0.32-1.17 | | 0.61* | 0.45-0.85 | 0.86 | 0.45-1.65 | | 0.66* | 0.48-0.92 | 0.89 | 0.46-1.74 | | 0.60* | 0.42-0.83 |
| **Income groups (yuan/month)** |  |  |  |  |  |  | |  |  |  |  | |  |  |  |  | |  |  |  |  | |  |  |
| 0-2000 | 1.00 |  | 1.00 |  | 1.00 |  | | 1.00 |  | 1.00 |  | | 1.00 |  | 1.00 |  | | 1.00 |  | 1.00 |  | | 1.00 |  |
| 2000-5000 | 0.64 | 0.34-1.19 | 1.32 | 0.89-1.95 | 1.53 | 0.93-2.52 | | 0.99 | 0.73-1.35 | 0.92 | 0.56-1.51 | | 0.97 | 0.72-1.32 | 1.11 | 0.69-1.79 | | 1.13 | 0.83-1.54 | 1.09 | 0.67-1.77 | | 1.02 | 0.74-1.40 |
| 5000-10000 | 0.68 | 0.37-1.24 | 1.04 | 0.68-1.58 | 1.13 | 0.71-1.80 | | 1.16 | 0.84-1.61 | 0.67 | 0.42-1.08 | | 1.11 | 0.81-1.53 | 1.04 | 0.67-1.63 | | 1.05 | 0.77-1.43 | 0.96 | 0.61-1.52 | | 0.99 | 0.72-1.37 |
| > 10000 | 0.95 | 0.50-1.78 | 1.46 | 0.87-2.46 | 0.93 | 0.58-1.49 | | 1.43 | 0.98-2.08 | 0.54* | 0.33-0.86 | | 1.22 | 0.85-1.75 | 1.26 | 0.81-1.98 | | 1.31 | 0.92-1.86 | 1.01 | 0.64-1.59 | | 1.16 | 0.81-1.66 |
| **Type of residence** |  |  |  |  |  |  | |  |  |  |  | |  |  |  |  | |  |  |  |  | |  |  |
| City | 1.00 |  | 1.00 |  | 1.00 |  | | 1.00 |  | 1.00 |  | | 1.00 |  | 1.00 |  | | 1.00 |  | 1.00 |  | | 1.00 |  |
| Town | 0.79 | 0.48-1.29 | 1.00 | 0.64-1.56 | 1.13 | 0.72-1.77 | | 1.18 | 0.84-1.65 | 0.69 | 0.45-1.05 | | 0.97 | 0.70-1.35 | 0.84 | 0.55-1.29 | | 1.00 | 0.72-1.39 | 0.68 | 0.43-1.07 | | 1.02 | 0.73-1.44 |
| Rural area | 0.93 | 0.48-1.82 | 0.68 | 0.39-1.18 | 1.14 | 0.64-2.03 | | 1.08 | 0.67-1.72 | 0.96 | 0.55-1.69 | | 0.78 | 0.49-1.25 | 1.33 | 0.76-2.33 | | 0.78 | 0.49-1.27 | 0.71 | 0.39-1.31 | | 0.80 | 0.48-1.33 |
| **Dementia contact** |  |  |  |  |  |  | |  |  |  |  | |  |  |  |  | |  |  |  |  | |  |  |
| Yes | 1.00 |  | 1.00 |  | 1.00 |  | | 1.00 |  | 1.00 |  | | 1.00 |  | 1.00 |  | | 1.00 |  | 1.00 |  | | 1.00 |  |
| No | 0.70* | 0.49-0.99 | 0.75* | 0.56-1.00 | 0.68* | 0.51-0.91 | | 0.74* | 0.60-0.92 | 0.82 | 0.62-1.07 | | 0.74* | 0.60-0.90 | 0.62* | 0.48-0.82 | | 0.69* | 0.57-0.84 | 0.55* | 0.42-0.73 | | 0.68* | 0.56-0.83 |
| Unclear | 1.18 | 0.73-1.91 | 0.91 | 0.62-1.34 | 0.74 | 0.52-1.08 | | 0.84 | 0.64-1.11 | 0.97 | 0.68-1.40 | | 0.86 | 0.66-1.12 | 0.77 | 0.54-1.10 | | 0.91 | 0.70-1.18 | 0.75 | 0.53-1.08 | | 1.01 | 0.78-1.31 |

**Supplementary Table 4. Sex differences in** **the association between** **demographic characteristics and the knowledge of protective factors for dementia.**

|  | **Exercise** | | | | **Social activity** | | | | **Intelligence games** | | | | **Reading** | | | | **Antihypertensive or hypolipidemic drugs** | | | |
| --- | --- | --- | --- | --- | --- | --- | --- | --- | --- | --- | --- | --- | --- | --- | --- | --- | --- | --- | --- | --- |
| **Gender** | **Male** | | **Female** | | **Male** | | **Female** | | **Male** | | **Female** | | **Male** | | **Female** | | **Male** | | **Female** | |
|  | AOR | 95% CI | AOR | 95% CI | AOR | 95% CI | AOR | 95% CI | AOR | 95% CI | AOR | 95% CI | AOR | 95% CI | AOR | 95% CI | AOR | 95% CI | AOR | 95% CI |
| **Age (years)** |  |  |  |  |  |  |  |  |  |  |  |  |  |  |  |  |  |  |  |  |
| < 40 | 1.00 |  | 1.00 |  | 1.00 |  | 1.00 |  | 1.00 |  | 1.00 |  | 1.00 |  | 1.00 |  | 1.00 |  | 1.00 |  |
| 40-65 | 0.52* | 0.34-0.81 | 0.65* | 0.45-0.93 | 0.74 | 0.54-1.03 | 0.92 | 0.67-1.25 | 0.42* | 0.31-0.58 | 0.54* | 0.40-0.72 | 0.74* | 0.56-0.98 | 0.84 | 0.65-1.08 | 0.80 | 0.46-1.39 | 0.72 | 0.48-1.10 |
| ≥ 65 | 0.34* | 0.13-0.88 | 0.45* | 0.21-0.94 | 0.45 | 0.19-1.08 | 0.66 | 0.32-1.38 | 0.61 | 0.26-1.45 | 0.42* | 0.21-0.83 | 0.40* | 0.17-0.92 | 0.61 | 0.32-1.14 | 4.03 | 0.76-21.33 | 0.23 | 0.03-1.84 |
| **Education level (years)** |  |  |  |  |  |  |  |  |  |  |  |  |  |  |  |  |  |  |  |  |
| Primary school or illiteracy (≤ 6) | 1.00 |  | 1.00 |  | 1.00 |  | 1.00 |  | 1.00 |  | 1.00 |  | 1.00 |  | 1.00 |  | 1.00 |  | 1.00 |  |
| Middle or high school (6-12) | 2.67 | 0.84-8.50 | 1.95 | 0.95-3.99 | 2.80 | 0.93-8.47 | 1.82 | 0.92-3.63 | 2.16 | 0.68-6.91 | 3.51* | 1.75-7.05 | 1.09 | 0.36-3.30 | 2.10 | 1.08-4.08 | NA | NA | 0.68 | 0.15-3.19 |
| College or university (12-16) | 3.02 | 0.91-10.04 | 3.92* | 1.82-8.44 | 4.53* | 1.46-14.06 | 3.27* | 1.58-6.76 | 3.83* | 1.18-12.47 | 10.71* | 5.15-22.24 | 2.10 | 0.69-6.44 | 5.24* | 2.64-10.43 | NA | NA | 0.93 | 0.20-4.36 |
| Postgraduate (≥ 16) | 6.23* | 1.68-23.04 | 4.81* | 2.02-11.43 | 7.12* | 2.19-23.16 | 6.29* | 2.80-14.12 | 5.17* | 1.53-17.54 | 17.14* | 7.68-38.25 | 2.62 | 0.83-8.25 | 7.15* | 3.43-14.86 | NA | NA | 0.86 | 0.18-4.17 |
| **Type of job** |  |  |  |  |  |  |  |  |  |  |  |  |  |  |  |  |  |  |  |  |
| Nonmanual | 1.00 |  | 1.00 |  | 1.00 |  | 1.00 |  | 1.00 |  | 1.00 |  | 1.00 |  | 1.00 |  | 1.00 |  | 1.00 |  |
| Manual | 0.78 | 0.42-1.44 | 0.55* | 0.35-0.86 | 0.78 | 0.49-1.25 | 0.74 | 0.50-1.11 | 0.65 | 0.41-1.02 | 1.09 | 0.72-1.63 | 0.66 | 0.43-1.01 | 0.74 | 0.53-1.04 | 0.82 | 0.31-2.20 | 0.73 | 0.36-1.47 |
| Retired | 0.56 | 0.26-1.19 | 0.50* | 0.32-0.78 | 0.87 | 0.43-1.77 | 0.84 | 0.54-1.30 | 0.32* | 0.16-0.63 | 0.71 | 0.48-1.03 | 0.80 | 0.41-1.53 | 0.83 | 0.58-1.19 | 0.37 | 0.08-1.81 | 0.94 | 0.46-1.94 |
| **Income groups (yuan/month)** |  |  |  |  |  |  |  |  |  |  |  |  |  |  |  |  |  |  |  |  |
| 0-2000 | 1.00 |  | 1.00 |  | 1.00 |  | 1.00 |  | 1.00 |  | 1.00 |  | 1.00 |  | 1.00 |  | 1.00 |  | 1.00 |  |
| 2000-5000 | 0.96 | 0.45-2.01 | 0.95 | 0.60-1.48 | 0.75 | 0.41-1.37 | 1.47 | 0.99-2.18 | 0.60 | 0.33-1.11 | 1.22 | 0.83-1.78 | 0.79 | 0.47-1.34 | 1.13 | 0.80-1.60 | 1.86 | 0.69-5.02 | 0.76 | 0.41-1.43 |
| 5000-10000 | 1.26 | 0.61-2.63 | 0.96 | 0.58-1.59 | 0.89 | 0.50-1.60 | 1.26 | 0.82-1.93 | 0.85 | 0.47-1.54 | 1.48 | 0.96-2.27 | 0.73 | 0.44-1.19 | 1.04 | 0.72-1.51 | 1.35 | 0.53-3.48 | 0.87 | 0.47-1.60 |
| > 10000 | 1.59 | 0.73-3.49 | 1.52 | 0.77-2.98 | 0.90 | 0.49-1.64 | 1.66 | 0.95-2.89 | 0.71 | 0.39-1.30 | 1.14 | 0.68-1.90 | 0.81 | 0.49-1.35 | 1.02 | 0.66-1.58 | 0.85 | 0.32-2.27 | 0.90 | 0.45-1.79 |
| **Type of residence** |  |  |  |  |  |  |  |  |  |  |  |  |  |  |  |  |  |  |  |  |
| City | 1.00 |  | 1.00 |  | 1.00 |  | 1.00 |  | 1.00 |  | 1.00 |  | 1.00 |  | 1.00 |  | 1.00 |  | 1.00 |  |
| Town | 1.58 | 0.79-3.16 | 1.19 | 0.71-1.99 | 1.16 | 0.70-1.93 | 0.72 | 0.48-1.09 | 1.15 | 0.70-1.87 | 1.04 | 0.68-1.58 | 1.08 | 0.69-1.68 | 1.00 | 0.69-1.45 | 0.26 | 0.06-1.09 | 0.68 | 0.30-1.52 |
| Rural area | 1.38 | 0.58-3.28 | 0.83 | 0.45-1.53 | 1.36 | 0.70-2.68 | 0.83 | 0.47-1.46 | 0.98 | 0.52-1.85 | 0.65 | 0.38-1.11 | 0.81 | 0.46-1.44 | 0.80 | 0.49-1.32 | 1.63 | 0.57-4.63 | 0.81 | 0.27-2.39 |
| **Dementia contact** |  |  |  |  |  |  |  |  |  |  |  |  |  |  |  |  |  |  |  |  |
| Yes | 1.00 |  | 1.00 |  | 1.00 |  | 1.00 |  | 1.00 |  | 1.00 |  | 1.00 |  | 1.00 |  | 1.00 |  | 1.00 |  |
| No | 0.91 | 0.57-1.46 | 0.79 | 0.56-1.12 | 0.82 | 0.58-1.16 | 0.74 | 0.55-1.01 | 0.86 | 0.62-1.20 | 0.88 | 0.66-1.18 | 0.67* | 0.49-0.90 | 0.92 | 0.72-1.17 | 0.44* | 0.25-0.77 | 0.51* | 0.35-0.76 |
| Unclear | 0.65 | 0.38-1.13 | 0.73 | 0.48-1.13 | 0.80 | 0.51-1.24 | 0.64* | 0.44-0.94 | 0.82 | 0.54-1.25 | 0.66* | 0.46-0.95 | 0.79 | 0.53-1.16 | 0.81 | 0.59-1.11 | 1.14 | 0.61-2.11 | 0.65 | 0.38-1.09 |
